# The Changes of Intrinsic Excitability of Pyramidal Neurons in Anterior Cingulate Cortex in Neuropathic Pain

**DOI:** 10.3389/fncel.2018.00436

**Published:** 2018-11-21

**Authors:** Zhilai Yang, Qilian Tan, Dan Cheng, Lei Zhang, Jiqian Zhang, Er-wei Gu, Weiping Fang, Xianfu Lu, Xuesheng Liu

**Affiliations:** Department of Anesthesiology, First Affiliated Hospital, Anhui Medical University, Hefei, China

**Keywords:** anterior cingulate cortex, intrinsic excitability, neuropathic pain, spontaneous excitatory postsynaptic currents, spike threshold, refractory period

## Abstract

To find satisfactory treatment strategies for neuropathic pain syndromes, the cellular mechanisms should be illuminated. Central sensitization is a generator of pain hypersensitivity, and is mainly reflected in neuronal hyperexcitability in pain pathway. Neuronal excitability depends on two components, the synaptic inputs and the intrinsic excitability. Previous studies have focused on the synaptic plasticity in different forms of pain. But little is known about the changes of neuronal intrinsic excitability in neuropathic pain. To address this question, whole-cell patch clamp recordings were performed to study the synaptic transmission and neuronal intrinsic excitability 1 week after spared nerve injury (SNI) or sham operation in male C57BL/6J mice. We found increased spontaneous excitatory postsynaptic currents (sEPSC) frequency in layer II/III pyramidal neurons of anterior cingulate cortex (ACC) from mice with neuropathic pain. Elevated intrinsic excitability of these neurons after nerve injury was also picked up, which was reflected in gain of input-output curve, inter-spike interval (ISI), spike threshold and Refractory period (RP). Besides firing rate related to neuronal intrinsic excitability, spike timing also plays an important role in neural information processing. The precision of spike timing measured by standard deviation of spike timing (SDST) was decreased in neuropathic pain state. The electrophysiological studies revealed the elevated intrinsic excitation in layer II/III pyramidal neurons of ACC in mice with neuropathic pain, which might contribute to central excitation.

## Introduction

Neuropathic pain is increasingly attracting the attentions of physicians and scholars worldwide. Although great efforts have been made to reveal the molecular mechanisms for this disease, there are still rarely effective treatment options for neuropathic pain patients currently (Finnerup et al., 2016). To find satisfactory treatment strategies for neuropathic pain syndromes, the cellular mechanisms should be illuminated, and treatment strategy for neuropathic pain on the cellular level may be a potential choice in the future.

Central sensitization is a generator of pain hypersensitivity, arising from different forms of structural and/or functional plasticity (Latremoliere and Woolf, 2009; Woolf, 2011; Huang et al., 2016). Synaptic plasticity in the nociceptive pathway plays an important role in central sensitization (Zhuo, 2014). In the past, researchers focused on the plastic changes in peripheral nociceptive nerve, dorsal root ganglia and spinal cord in different forms of pain (Latremoliere and Woolf, 2009). Nowadays, clinical trials and animal experiments reveal the importance of neuronal plasticity in cerebral cortex in neuropathic pain state, including the anterior cingulate cortex (ACC), the somatosensory cortex, the prefrontal cortex and the insular cortex (LaGraize et al., 2006; Zhuo, 2014). ACC is an important component in limbic system which is related to emotion, memory and behavior (Bush et al., 2000). Several studies have shown that ACC is involved in encoding the emotional aspect of pain. Frontal cingulumotomy including the ACC in patients with pain could relief pain syndrome by altering the emotional responses to pain (Foltz and White, 1962; Bushnell et al., 2013). Animal studies by destruction of neurons also revealed that neurons in ACC were necessary for the emotional aspect of pain (Johansen et al., 2001). Functional imaging with positron emission tomography or fMRI provided direct evidence linking ACC activity with pain affect in patients or normal human volunteers (Rainville et al., 1997; Bushnell et al., 2013). All these studies emphasize the importance of ACC in pain. So, we choose ACC as our target brain area in the current study.

A typical neuron receives thousands of synaptic inputs from presynaptic neurons (van Vreeswijk and Sompolinsky, 1996). If the summated postsynaptic currents exceed spike threshold, a single spike will be generated. So, the excitability of a neuron depends on two components, the synaptic inputs and the intrinsic excitability. Previous studies focused on the synaptic inputs in neuropathic pain (Zhao et al., 2006; Gong et al., 2010; Zhuo, 2014). But little is known about the changes of neuronal intrinsic excitability in the pain pathway after nerve injury. However, neuronal intrinsic excitability indeed changed during some pathological status, such as Alzheimer’s disease (Brown et al., 2011), Angelman syndrome (Kaphzan et al., 2011), seizer (Villeneuve et al., 2000), MELAS syndrome (Iizuka et al., 2002), or by hydrogen peroxide (Ohashi et al., 2016) and Pumilio-2 (Driscoll et al., 2013). Here, we studied the intrinsic excitability of layer II/III pyramidal neurons from ACC in neuropathic pain, including input-output curve, inter-spike interval (ISI), spike threshold and Refractory period (RP). Besides firing rate related to neuronal intrinsic excitability, spike timing is another information carrier in the central nervous system (Schneidman et al., 1998; Tiesinga et al., 2008). We studied the precision of spike timing by the standard deviation of spike timing (SDST) in this study (Chen et al., 2006a). The electrophysiological studies revealed that intrinsic excitability of layer II/III pyramidal neurons in ACC was increased after nerve injury, which might contribute to the central excitation.

## Materials and Methods

### Animals

Male C57BL/6J mice were purchased from the Laboratory Animal Center of Anhui Medical University, and were fed with standard laboratory diet and tap water in climate- and light-controlled conditions under 12-h light-dark cycles. The mice were housed for at least 1 week prior to the experiments and 8 week at the time of operation (Smith et al., 2013). This study was carried out in accordance with the recommendations of Animal Care and Use Committee of Anhui Medical University. The protocol was approved by the Ethics Committees of Anhui Medical University. Forty mice were used in this study, 20 for the recording of spontaneous excitatory postsynaptic currents (sEPSC) and 20 for the recording of intrinsic excitability.

### The Spared Nerve Injury (SNI) Model of Neuropathic Pain

Spared nerve injury (SNI) and sham surgery were performed under 1% pentobarbital anesthesia (50 mg/kg ip) according to the operative methods described previously (Decosterd and Woolf, 2000; Shields et al., 2003). For mice in SNI model group, axotomy and ligation of the tibial and common peroneal branches were performed after exposing the sciatic nerve and its three terminal branches, and leaving the sural nerve intact. Muscle and skin layers were carefully closed. For mice in control group (sham-operated), the nerves were only exposed without axotomy and ligation, and the muscle and skin were carefully closed. Caution was taken not to stretch the intact sural nerve during the surgery. Mechanical withdrawal threshold was measured by an Electronic Von Frey Apparatus (Martinov et al., 2013). After acclimation for 15 min in chambers prior to the measurement, the hind paw is stimulated with the probe. Increasing the pressure gradually, the pressure to induce nociceptive response behaviors, such as hind paw retraction, hind paw licking, or four-paw jumping, is defined as mechanical withdrawal threshold. The average of three measurements for each mouse is taken as mechanical withdrawal threshold. Mechanical withdrawal thresholds were measured on the day before surgery, as well as 7 days after surgery (Decosterd and Woolf, 2000).

### Electrophysiology Study

The coronal brain slices containing ACC (400 μm) were prepared in mice 1 week after operation. Mice were anesthetized with 1% pentobarbital (50 mg/kg ip) and decapitated with a guillotine (Yang et al., 2014, 2017). The slices were cut with vibratome in the oxygenated (95% O_2_ and 5% CO_2_) ACSF (124 mM NaCl, 3 mM KCl, 1.2 mM NaH_2_PO_4_, 26 mM NaHCO_3_, 0.5 mM CaCl_2_, 4 mM MgSO_4_, 10 mM dextrose and 5 mM HEPES; pH 7.35) at 4°C (Yang et al., 2017). The slices were held in oxygenated ACSF (126 mM NaCl, 2.5 mM KCl, 2 mM MgSO_4_, 2 mM CaCl_2_, 26 mM NaHCO_3_, 1.25 mM NaH_2_PO_4_ and 25 mM dextrose; pH 7.35) at 25°C for at least 1 h. A slice was transferred to a submersion chamber (Warner RC-26 G) that was perfused with the oxygenated ACSF at 32°C. Layer II/III pyramidal neurons in ACC were recorded by whole-cell patch clamp under DIC optics (Olympus BX51WI). Pipettes were filled with solutions containing (in mM) 150 K-gluconate, 5 NaCl, 5 HEPES, 0.4 EGTA, 4 Mg-ATP, 0.5 Tris-GTP and 5 phosphocreatine (pH 7.35 adjusted by 2M KOH, 295–305 mOsmol). The junction potential for these solutions was 18, and we had not corrected for it. All patch clamp recordings were sampled at 50 kHz and low-pass filtered at 10 kHz. Series resistance was compensated. If the changes of series resistance during the experiment were larger than 10%, we would exclude the data (Kaphzan et al., 2011).

**Figure 1:**
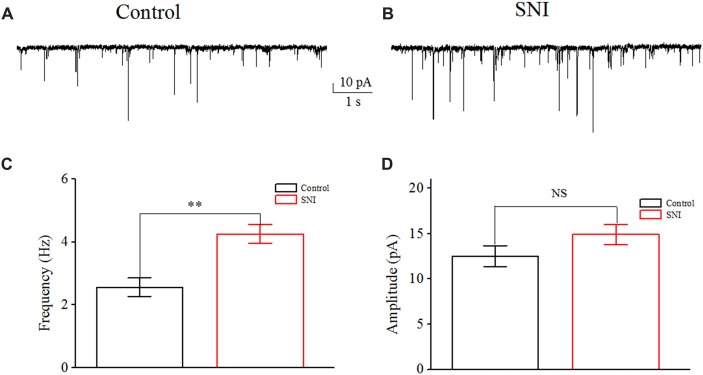
Increased spontaneous excitatory postsynaptic current (sEPSC) in layer II/III pyramidal neurons of anterior cingulate cortex (ACC) after nerve injury. **(A,B)** Representative sEPSC from these neurons in Control group and in spared nerve injury (SNI) group. **(C)** The mean frequency of sEPSC recorded from these neurons in Control group (*n* = 10) and in SNI group (*n* = 10). **(D)** The mean amplitude of sEPSC recorded from these neurons in Control group (*n* = 10) and in SNI group (*n* = 10). NS, no significant difference. **p < 0.01.

In order to illustrate the changes of excitatory synaptic transmission in neuropathic pain state, the sEPSC of layer II/III pyramidal neurons in ACC were recorded. These cells were identified by the cellular type and their firing characteristics (adaptation) as previous study (Zhao et al., 2005; Qiu et al., 2014). Layer II/III pyramidal neurons in ACC have different firing pattern *in vivo* and *in vitro*: regular spiking (RS), intermediate (IM) and intrinsic bursting (IB; Cao et al., 2009; Koga et al., 2010). We do not observe any firing profile conversion after nerve injury. sEPSC was recorded at a holding potential of −70 mV for 5 min, in the continuous presence of 20 μM bicuculline (antagonist of GABAA receptors). We also administrated CNQX (10 μM) and D-AP5 (40 μM) to certify the sEPSC at the end of the experiment. Except for sEPSC recordings, the other recordings about intrinsic excitation were made at the resting membrane potential, in the absence of bicuculline. sEPSC events were automatically detected by template matching using ClampFit 10 (Axon Instruments; Matta et al., 2013). The frequency and amplitude in Figure 1 are the mean values of all the sEPSC events.

We then studied the neuronal intrinsic excitability of layer II/III pyramidal neurons in ACC. The gain of input-output curves and ISIs were investigated to indicate the spiking ability of a neuron. The input-output curve is conducted by gradually increasing the stimulus intensity of depolarizing pulse (1,000 ms; Yang et al., 2017), and the first amplitude of the stimulus current is 10 pA, increasing by 20 pA. The gain of input-output curve is defined as the slope of the linearly fitted curve (Zhang and Arsenault, 2005; Thurley et al., 2008). ISI was analyzed by evoking a spike train by somatic depolarizing pulse (1,000 ms) at the intensity of spike threshold. ISI is the time duration between two neighboring spikes (Chen et al., 2006a). The first ISI in Figure 3 refers to the ISI between the first and the second spike, and the steady-state ISI refers to the mean ISI in the steady-state firing.

**Figure 2:**
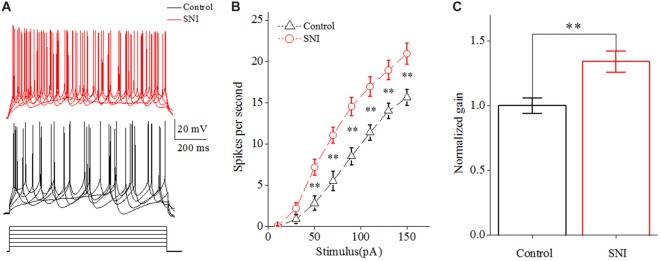
Increased spiking ability in layer II/III pyramidal neurons of ACC after nerve injury. **(A)** Representative spike train from these neurons in SNI group (red, top) and in Control group (black, middle); depolarizing pulse (1,000 ms) as stimulus waveform (black, bottom). **(B)** The increased gain of input-output curve (*n* = 16, Control group; *n* = 17, SNI group). **(C)** The normalized gain in the two groups. **p < 0.01.

**Figure 3:**
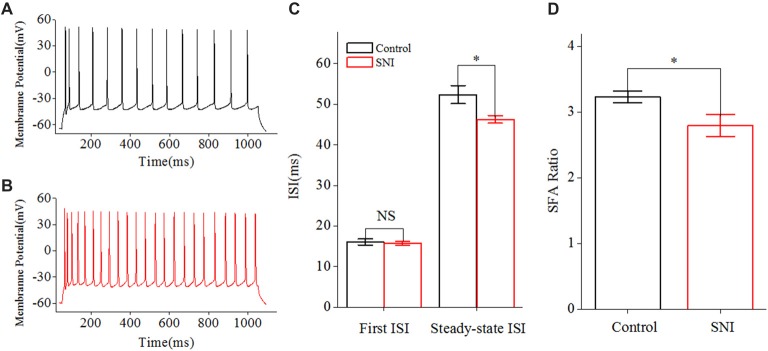
Decreased inter-spike interval (ISI) in layer II/III pyramidal neurons of ACC after nerve injury. **(A,B)** Representative spike train from these neurons in Control group (**A**, black) and in SNI group (**B**, red). **(C)** First and Steady-state ISI (*n* = 16, Control group; *n* = 17, SNI group). **(D)** Decreased spike frequency adaptation (SFA) ratio after nerve injury. NS, no significant difference. *p < 0.05.

The neuronal intrinsic excitability also includes spike threshold for initiating a spike and RP after a spike. The spike threshold in this study referred to the current threshold, and was detected by increasing stimulus intensity until inducing a spike at 50% chance by somatic depolarizing pulse (10 ms; Chen et al., 2006a). The spike threshold reflects the difficulty degree for a neuron to turn synaptic inputs into spikes. RP is measured by injecting paired depolarizing pulses (amplitude: four times of spike threshold; time: 3 ms) into a neuron to induce a pair of spikes. By changing inter-pulse interval, we define RP as the time duration from a complete spike to its subsequent spike at 50% probability (Chen et al., 2006a). It should be noted that the RP investigated in this study was the absolute RP. The RP determined the theoretically maximal firing rate for a neuron.

Spike timing plays an important role in temporal encoding and time coding for neuronal information processing (Borst and Theunissen, 1999). Precision is one important index for spike timing. We studied the precision of spike timing by the SDST. SDST was analyzed by evoking spike trains with somatic depolarizing pulses (amplitude: spike threshold; time: 1,000 ms). The first SDST in Figure 6 refers to the SDST of the first spike, and the steady-state SDST refers to the mean SDST in the steady-state firing. The increased SDST reflected decreased precision of spike timing, and vice versa.

**Figure 4:**
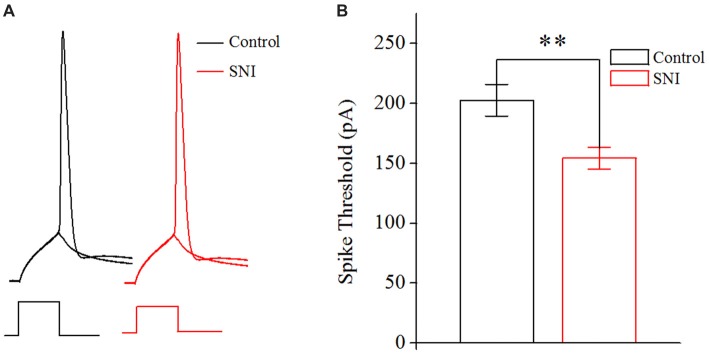
Decreased spike threshold in layer II/III pyramidal neurons of ACC after nerve injury. **(A)** Representative spike threshold to induce spike at 50% chance (black in Control group; red in SNI group). **(B)** Decreased spike threshold after nerve injury (*n* = 16, Control group; *n* = 17, SNI group). **p < 0.01.

**Figure 5:**
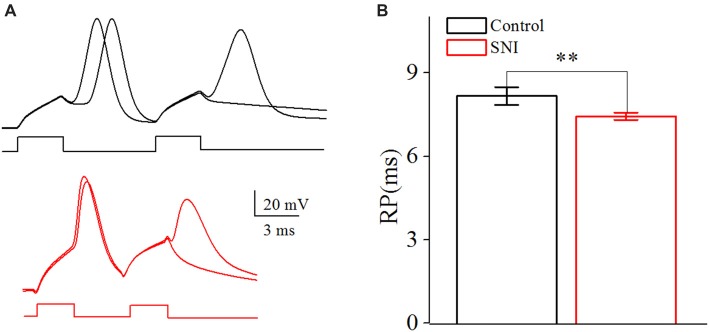
Decreased spike Refractory period (RP) in layer II/III pyramidal neurons of ACC after nerve injury. **(A)** Representative spike RP in Control group (black, top) and in SNI group (red, bottom). **(B)** Decreased spike RP after nerve injury (*n* = 16, Control group; *n* = 17, SNI group). **p < 0.01.

**Figure 6:**
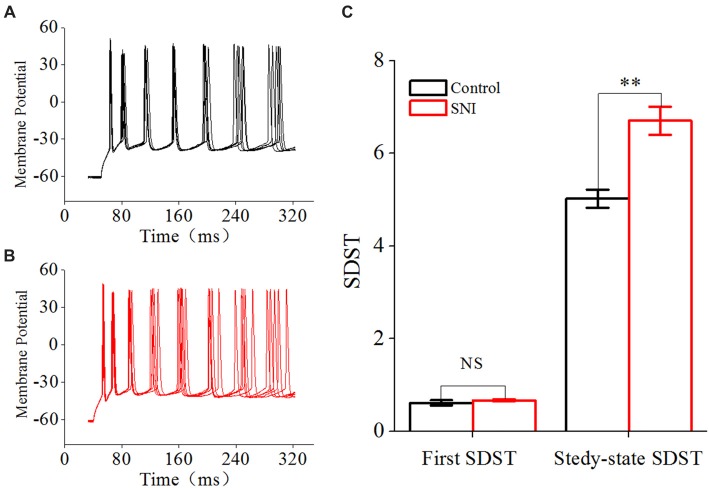
Decreased spike timing precision in layer II/III pyramidal neurons in ACC after nerve injury. **(A,B)** Representative spike train in Control group (**A**, black) and in SNI group (**B**, Red). Note the different dispersion degree for spikes in the two groups. **(C)** First and Steady-state standard deviation of spike timing (SDST; *n* = 16, Control group; *n* = 17, SNI group). NS, no significant difference. **p < 0.01.

### Statistical Analysis

Results were expressed as Mean ± SEM. Statistical comparisons under different conditions were done by *t*-test, or two-way ANOVA (repeated measurements). In all cases, differences were considered statistically significant at *p < 0.05 and **p < 0.01.

## Results

The mechanical withdraw threshold was measured to ensure the success of SNI surgery. As mentioned in other researches, the decreased mechanical withdraw threshold indicated allodynia-like behavior in SNI group (normalized withdrawal threshold: 1 ± 0.07 before surgery vs. 0.12 ± 0.02 7 days after surgery, *n* = 10, *P* < 0.01). But, there was no significant change of mechanical withdraw threshold before and after operation in Control group (normalized withdrawal threshold: 0.99 ± 0.05 before surgery vs. 0.98 ± 0.04 7 days after surgery, *n* = 10, *P* = 0.87). We then studied the neuronal excitability of layer II/III pyramidal neurons in ACC by patch clamp recordings. Excitatory synaptic transmission was one important component of neuronal excitability. So, we firstly studied the difference of sEPSC of these neurons between Control and SNI groups.

### Increased sEPSC Frequency in Layer II/III Pyramidal Neurons of ACC in Mice With Neuropathic Pain

Increased excitatory synaptic transmission in pain pathway has been invested in some pathological pain state. We measured the frequency and amplitude of sEPSC in layer II/III pyramidal neurons of ACC. Typical sEPSC traces in these neurons were shown (Figures 1A,B). A significant increase in sEPSC frequency was detected in mice with neuropathic pain compared with controls (Control: 2.56 ± 0.29 Hz; SNI: 4.26 ± 0.29 Hz; *P* < 0.01, independent sample *t*-test; Figure 1C). But no significant difference in the amplitude of sEPSCs was detected between the two groups (Control: 12.52 ± 1.16 pA; SNI: 14.93 ± 1.10 pA; *P* = 0.15, independent sample *t*-test; Figure 1D). The increased excitatory synaptic transmission means that these neurons receive more excitatory inputs in neuropathic pain state, thus may contribute to neuronal hyperexcitability in pain. On the other hand, neuronal excitability also depends on neuronal intrinsic excitability, but its role in neuropathic pain remains elusive. Then, the intrinsic excitability of these neurons was revealed by whole-cell patch clamp recordings. Sixteen neurons were recorded from 10 mice in Control group and 17 neurons were recorded from 10 mice in SNI group.

### Increased Spiking Ability of Layer II/III Pyramidal Neurons in ACC in Mice With Neuropathic Pain

The gain of input-output curve (the slope of the curve) was measured to illustrate the spiking ability for a neuron (Zhang and Arsenault, 2005; Thurley et al., 2008). By taking spike frequency as a function of the stimulus intensity, we could obtain input-output curve. The gain of input-output curve was increased in these neurons after nerve injury (Figures 2A,B; *P* < 0.01, two-way ANOVA (repeated measurements)). The normalized gain was 1 ± 0.06 in Control group and 1.34 ± 0.08 in SNI group (Figure 2C). In order to find out the foundation for the increased gain in neuropathic pain, we studied the ISI. ISI is the time duration between two successive spikes in a spike train. The first ISI was not changed after nerve injury, but the steady-state ISI was significantly decreased in SNI group compared with Control group (Figure 3C, *P* = 0.02, independent sample *t*-test). The first ISI was 16.07 ± 0.76 ms in Control group and 15.77 ± 0.53 ms in SNI group. The steady-state ISI was 52.36 ± 2.22 ms in Control group and 46.26 ± 0.91 ms in SNI group. We also measured spike frequency adaptation (SFA) ratio, which equaled the steady-state ISI divided by the first ISI (Fernandez et al., 2011). The SFA ratio was decreased in mice with neuropathic pain (Figure 3D, control 3.23 ± 0.09, SNI 2.8 ± 0.17; (*P* = 0.03, independent sample *t*-test)), which might contribute to the decreased spiking ability. From these results we can come to the conclusion that the spiking ability of layer II/III pyramidal neurons in ACC is increased in neuropathic pain. The intrinsic excitability also includes threshold for initiating a spike and RP after a spike. We then compared the spike threshold and RP of these neurons between the two groups.

### Decreased Spike Threshold in Layer II/III Pyramidal Neurons of ACC in Mice With Neuropathic Pain

In order to illustrate the changes of intrinsic excitability in neuropathic pain, we studied the spike threshold of layer II/III pyramidal neurons in ACC. Postsynaptic currents from thousands of presynaptic neurons summate to reach spike threshold, and then a spike will generate. So, the spike threshold reflects how easy a neuron turns synaptic input into spikes. The spike threshold (the least current to induce a spike) was decreased in neuropathic pain (Figures 4A,B, *P* < 0.01, independent sample *t*-test). The spike threshold was 202.65 ± 13.24 pA in Control group and 154.25 ± 9.09 pA in SNI group. The decreased spike threshold means that neurons will need less excitatory synaptic input to induce a spike, i.e., increased intrinsic excitability for these neurons in neuropathic pain.

### Decreased Refractory Period in Layer II/III Pyramidal Neurons of ACC in Mice With Neuropathic Pain

The RP referred to the shortest interval between action potentials at a given strength of the testing stimulus (Farmer et al., 1960), which determined the theoretically maximal firing rate for a neuron. By changing the inter-pulse interval of the depolarizing pulses, we defined RP as the time duration from a spike to a subsequent spike at 50% of firing probability (Chen et al., 2006a; Figure 5A). The RP was decreased from 8.16 ± 0.32 ms in Control group to 7.44 ± 0.13 ms in SNI group (Figure 5B, *P* = 0.04, independent sample *t*-test). The decreased spike threshold and RP reflect the elevated intrinsic excitability for these neurons in neuropathic pain. The passive properties of these neurons were also considered. There was no significant difference in the resting membrane potential (Control: −61.2 ± 1.1; SNI −60.7 ± 1.2; *P* = 0.76) and cell resistance (Control: 172.2 ± 11.6; SNI: 187.6 ± 14.0; *P* = 0.41) between the two groups. And AHP had little change after nerve surgery (Control: 10.75 ± 0.72 mV; SNI 11.13 ± 0.92 mV; *P* = 0.75). Afterhyperpolarization (AHP) was defined as the differences between the peak hyperpolarizing voltage deflection following a spike and the voltage threshold (Ohashi et al., 2016).

### Reduced Spike Timing Precision in Layer II/III Pyramidal Neurons of ACC in Mice With Neuropathic Pain

Besides spike rate related to neuronal spiking ability, spike timing was another important information carrier, and might play an important role in building neuronal code or information processing (Mainen and Sejnowski, 1995; Stanley, 2013). Spike timing precision could change in some physiological and pathological states (Foffani et al., 2007; Orduz et al., 2013). We used SDST to reflect spike timing precision in this study, which was calculated from 20 traces of spike train induced by repetitive identical stimuli. The first SDST was not changed after nerve injury, but the steady-state SDST was significantly increased in SNI group compared with Control group (Figure 6C, *P* < 0.01, independent sample *t*-test). The first SDST was 0.62 ± 0.06 in Control group and 0.67 ± 0.03 ms in SNI group. The steady-state SDST was 5.02 ± 0.2 in Control group and 6.7 ± 0.3 in SNI group. These results revealed that spike timing precision was decreased in neuropathic pain. The decreased spike timing precision might participate in information processing in neuropathic pain.

## Discussion

As mentioned in previous studies, excitatory synaptic transmission was enhanced during pain. In layer II/III pyramidal neurons of ACC in mice with neuropathic pain, we also found increased sEPSC frequency. More importantly, we measured the intrinsic excitability of these neurons in neuropathic pain. The spiking ability represented by gain of input-output curve and ISI was elevated in these neurons after nerve injury. And it might attribute to the decreased spike threshold and RPs in neuropathic pain. On the other hand, the precision of spike timing in these neurons was declined in neuropathic pain, which might affect the neuronal code or information processing. A recent study also revealed elevated intrinsic excitability in the somatosensory cortex in tibial nerve injury (TNI) and tSCI mice models (Xiong et al., 2017). This study focused on the homeostatic plasticity in response to lesion-induced somatosensory deprivation and activity loss, which was revealed to induce the elevated intrinsic excitability. We studied the intrinsic excitability of layer II/III pyramidal neurons in ACC from SNI mice model. Spike rate and spike timing were two important information carrier in the central nervous system (Prescott and Sejnowski, 2008). So, we focused on neuronal intrinsic excitation, which was related to neuronal spiking ability and spike timing precision, in this study. In our opinion, this is the first systematic study about intrinsic excitability and spike timing precision in mice with neuropathic pain, although sparse evidence about intrinsic excitability has been mentioned in pain state in previous studies.

Neurons convey information through complex patterns of spikes which are driven by integrated synaptic inputs. Synaptic transmission is one of the most important parts in neuronal information processing. Many studies have been carried out focusing on synaptic transmission in ACC during pain (Gong et al., 2010; Li et al., 2014; Zhuo, 2014; Bliss et al., 2016). And some researchers even take cortical plasticity in ACC (Zhuo, 2014) or in insular cortex (Zhuo, 2016), as a new endpoint measurement for chronic pain. As mentioned in other pain pathway (Zhuo, 2016, 2017), we found that the excitatory postsynaptic currents of layer II/III pyramidal neurons in ACC were increased in neuropathic pain. The increased excitatory synaptic inputs certainly induce more spikes in these neurons, and thus contribute to neuronal hyperexcitability. Besides enhanced excitatory synaptic transmission in neuropathic pain, reduced inhibitory synaptic transmission might also contribute to central sensitization (Gong et al., 2010). Referring to presynaptic or postsynaptic mechanisms underlying hyperexcitability, a previous study has revealed enhanced presynaptic neurotransmitter release during chronic pain by measuring the paired pulse ratio and miniature EPSC (Zhao et al., 2006).

The neuronal excitability depends both on the synaptic inputs and on the intrinsic properties (Beck and Yaari, 2008). In the present study, we also measured neuronal spiking ability by gain of input-output curve and ISIs, and found that spiking ability of layer II/III pyramidal neurons in ACC was increased in neuropathic pain. Neuronal input-output properties determine neuronal response, and the modulation of its gain is an important computational feature (Devanne et al., 1997). Gain modulation of pyramidal neurons might arise from noisy input (Arsiero et al., 2007), gamma rhythms (Sohal et al., 2009) and neurotransmitter application (Zhang and Arsenault, 2005; Thurley et al., 2008). Gain modulation in ACC might be the basis for central sensitization in neuropathic pain. In order to evaluate the intrinsic excitability in mice with neuropathic pain, we also measured spike threshold and RP. The spike threshold distinguished suprathreshold depolarization from subthreshold (Yi et al., 2015). And it was the foundation of neuronal input-output properties. The changes of spike threshold might induce gain modulation (Azouz and Gray, 2003). The RP after each spike reflected the time latency to elicit another spike by outside stimulus, and it underlay neuronal spiking ability and spike timing (Chen et al., 2006a). The decreased spike threshold and RP might cause gain modulation, participating in central sensitization in neuropathic pain.

With regard to mechanisms for the elevated intrinsic excitability, voltage-gated sodium channel (VGSC) might be preferred. VGSC was the hotspot in neuropathic pain studies, especially sodium channel subtypes expressed in the peripheral nervous system or spinal cord, such as Nav1.7, Nav1.8 and Nav1.9 (Lampert et al., 2010). Nowadays, sodium channel subtypes expressed in the cerebral cortex, such as Nav1.2 and Nav1.6, were also believed to participate in neuropathic pain (Priest et al., 2004; Liao et al., 2010; Xie et al., 2013). The mRNA expression of Nav1.1, Nav1.2, Nav1.6, Navb1 and Navb3, was significantly increased by paclitaxel treatment (Masocha, 2016). Cannabinoid could alleviate neuropathic pain by inhibiting the functions of VGSCs (Okura et al., 2014). Besides its role in pain, VGSC was also the most important candidate underlying intrinsic excitability (Chen et al., 2006b; Goldfarb et al., 2007). The subcellular distribution and biophysical properties of VGSC determined intrinsic excitability of neurons (Goldfarb et al., 2007). So, the roles of VGSC in the elevated intrinsic excitability during pain will be the goal of our future research. And this might help us to develop drugs for neuropathic pain.

Besides spike rate related to neuronal intrinsic excitability, spike timing was believed to be another important information carrier in the central nervous system (Schneidman et al., 1998; Tiesinga et al., 2008). Spike timing was the basis for temporal encoding and time coding during neuronal information processing (Borst and Theunissen, 1999). Precision was one important index for spike timing, and reduced spike timing precision was related to some pathological state (Foffani et al., 2007). Peripheral injuries increased the spike timing jitter in neurons of ACC *in vivo* (Li et al., 2014). We also found reduced spike timing precision in neuropathic pain by measuring SDST in the two groups. The roles of reduced spike timing precision in neuropathic pain remained unknown. And this might be an important issue for revealing the pathological state of neuropathic pain.

In summary, our research demonstrated increased intrinsic excitability and reduced spike timing precision in layer II/III pyramidal neurons of ACC in mice with neuropathic pain. The change in spike frequency and spike timing precision has been discussed in previous studies (Prescott et al., 2006; Prescott and Sejnowski, 2008). Steven A Prescott and collaborator revealed that a pyramidal neuron encoded time-averaged input with firing rate at low conductance state, but encoded transient inputs with precisely timed spikes at high conductance state. So, the shift of cell conductance switched the operational mode from integration to coincidence detection (Prescott et al., 2006). The spike-rate coding and spike-time coding, were all affected by SFA (Prescott and Sejnowski, 2008). Calcium-activated K current, which underlay SFA, improved spike-rate coding at the cost of spike-time coding. Voltage-activated M-type K current (IM), which represented another mechanism for SFA, improved spike-time coding but destroyed spike-rate coding. So, more in-depth researches are needed to reveal the mechanisms for the changes of spike-rate coding and spike-time coding in neuropathic pain.

## Author Contributions

ZY and XuL designed the research and wrote the article. ZY, QT, DC, LZ and JZ performed the research and analyzed the data. EG, WF and XiL revised the manuscript.

## Conflict of Interest Statement

The authors declare that the research was conducted in the absence of any commercial or financial relationships that could be construed as a potential conflict of interest.
